# Effect of High-Pressure Homogenization and Wall Material Composition on the Encapsulation of Polyunsaturated Fatty Acids from Fish Processing

**DOI:** 10.3390/molecules30071434

**Published:** 2025-03-24

**Authors:** Ioanna Semenoglou, Maria Katsouli, Maria Giannakourou, Petros Taoukis

**Affiliations:** Laboratory of Food Chemistry and Technology, School of Chemical Engineering, National Technical University of Athens, 15780 Athens, Greece; isemen@chemeng.ntua.gr (I.S.); mkatsouli@chemeng.ntua.gr (M.K.); mgian@chemeng.ntua.gr (M.G.)

**Keywords:** fish oil, nanoemulsions, high-pressure homogenization, encapsulation, Arabic gum, sodium alginate, maltodextrin

## Abstract

Fish oil, a rich source of omega-3 polyunsaturated fatty acids (PUFA), is a vital nutritional component, but considering its susceptibility to oxidation, it could benefit from an effective encapsulation system. This study aims to optimize high-pressure homogenization (HPH) parameters (pressure, number of passes) and wall material composition to maximize the encapsulation efficiency (EE) of fish oil, using different concentrations of maltodextrin with Arabic gum or sodium alginate. Key metrics such as emulsion droplet size, encapsulation efficiency, color, and oxidation in the final freeze-dried product were evaluated. Optimal values were achieved at 60 MPa, resulting in the lowest mean droplet diameter (369.4 ± 3.8 nm) and narrow distribution (0.197 ± 0.011) of the fish oil micelles prepared with a mixture of Tween80 and sodium caseinate as an emulsifier, without significant oxidation after four cycles of homogenization, while 80 MPa led to the highest EE (up to 95.6%), but increased oxidation. The combination of 10% *w*/*w* Arabic gum or 1% *w*/*w* sodium alginate with 20% *w*/*w* maltodextrin achieved the highest EE (79.1–82.9%) and whiteness index (82.5–83.0), indicating neutral-colored well-encapsulated fish oil without oxidation, which is desirable for product stability. Selecting optimal HPH conditions and wall material is crucial for the encapsulation efficiency and oxidation stability of omega-3 PUFA delivered in dehydrated forms.

## 1. Introduction

The Food and Agriculture Organization of the United Nations/the World Health Organization (FAO/WHO) recommend the reduction of saturated fatty acids intake while promoting the increased consumption of polyunsaturated fatty acids (PUFA), aiming to reduce the risk of chronic diseases, including coronary heart disease. The recommendation is to consume <10% energy intake as saturated fatty acids and to consume 6–11% energy intake as PUFA. Studies support that docosahexaenoic acid (DHA, C22:6 n-3) and eicosapentaenoic acid (EPA, C20:5 n-3) can provide a wide range of health advantages, such as hypotriglyceridemic and anti-inflammatory effects. The recommended intake of omega-3 PUFA varies and depends on different parameters, such as age, pregnancy status, overall health status, and specific physiological needs. Based on European Food Safety Authority (EFSA), an average intake of 250–500 mg/day of DHA + EPA is recommended for European adults in relation to cardiovascular health. The National Institutes of Health (NIH) recommends an adequate intake of 1.6 and 1.1 g of omega-3 PUFAs per day for men and women, respectively, while the respective value for children up to 8 years old is 0.9 g/day. For pregnant and lactating women, the intake should be 1.4 g and 1.3 g, respectively [1,2,3].

Incorporating PUFA into many functional foods and/or beverage products offers an opportunity to increase their intake. However, this process faces several challenges, because DHA and EPA are characterized by poor water-solubility, an unpleasant odor and flavor, low oxidative stability, and low bioavailability [4]. Several types of liquid (nanoliposomes, nanoemulsions) or solid (nanoparticles, nanogels, microencapsulation powders) delivery systems have been proposed to overcome these drawbacks [5,6]. Spray drying, spray chilling, freeze drying (also known as lyophilization), and fluidized bed technology are the most frequently proposed techniques in order to microencapsulate oil into powders. The spray-drying method is the most commonly used encapsulation process in the food industry as it is convenient, affordable, and produces amorphous (non-crystalline) powders. These powders can improve the solubility and bioavailability of encapsulated materials [7,8,9]. However, some heat-sensitive materials may not be suitable for spray drying due to the high temperatures involved in the process. The freeze-drying method is an excellent alternative to encapsulate thermally sensitive lipophilic bioactive compounds such as fish oil. Freeze drying is a gentle process that minimizes the exposure of sensitive core materials to heat and oxidation, making it suitable for encapsulating pharmaceuticals, probiotics, enzymes, and other sensitive biologically active substances [10,11]. The porous microstructure formed during freeze drying can help control the release of the encapsulated material, allowing for gradual release over time [7].

Important steps in the food microencapsulation process include the selection of an appropriate homogenization and drying process, along with choosing a suitable emulsifier and wall materials. Researchers suggest that combining proteins and polysaccharides that serve as both a carrier matrix and an emulsifier is the ideal technique to emulsify fish oil [12,13]. Thus, it is necessary to determine which protein–polysaccharide combinations and drying technique are the optimal choice for the development of fish oil (PUFA) nanoemulsion powders [14]. Moreover, it is essential to develop the nanoemulsion feed with desirable properties, i.e., small droplet size, a narrow size distribution, and low viscosity for freeze drying. These properties help produce powders with enhanced encapsulation efficiency and oxidation stability, ensuring consistent and reliable results [15]. In freeze drying, the size of these particles is influenced by the initial droplet size. Controlling the droplet size can help to regulate the final particle size, which is crucial for encapsulation applications. Smaller droplets are less prone to agglomeration, which can lead to the formation of larger particles and non-uniform encapsulation. Due to the very small droplet size of nanoemulsions (less than 500 nm), the produced powders after freeze drying exhibit a uniform distribution of the encapsulated bioactive compounds and high encapsulation efficiency. The droplet size of the emulsion feed is important for freeze-drying encapsulation efficiency, particularly when encapsulating active ingredients. Smaller droplets provide a larger total surface area compared to larger droplets, allowing for more efficient and uniform freeze-dried powders. The increased surface area allows for faster drying and better preservation of the encapsulated materials. Additionally, smaller droplets have a higher surface-to-volume ratio, which also promotes efficient heat and mass transfer during the freeze-drying process. This helps to prevent the encapsulated materials from being exposed to heat and moisture for extended periods, which can be detrimental to their stability [16,17].

Small molecule surfactants like Tween, span, etc., and polysaccharides, proteins, and modified starches are two types of often utilized emulsifiers. When it comes to stabilizing oil-in-water nanoemulsions, mixtures of small molecule surfactants, proteins, and polysaccharides (pectin, alginate, Arabic gum) are proposed. Proteins and polysaccharides can contribute to stability through a combination of steric stabilization and electrostatic interactions when applicable. Νon-ionic surfactants, such as Tween 80, stabilize emulsions by reducing interfacial tension and providing steric stabilization. Even though non-ionic surfactants do not directly participate in charge-based interactions, they can modify interfacial properties, facilitating better adsorption of proteins and polysaccharides onto the oil droplet surface, thereby improving emulsion stability. The food industry has widely adopted proteins and polysaccharides as natural biopolymers [14,18]. Due to their widespread commercial availability and good film-forming and amphiphilic qualities, numerous casein proteins have been extensively studied as wall materials for encapsulation systems. Casein proteins are widely used because they can successfully lower the surface tension at the oil–water interface, are quickly absorbed, and create thick interfacial coatings during emulsification. Caseins prevent oxidation of emulsified oils, primarily by forming a protective interfacial layer around oil droplets, limiting their direct exposure to pro-oxidants such as oxygen and transition metals. Moreover, their iron chelating properties can influence lipid oxidation by sequestering free iron ions [19,20,21].

The field of PUFA microencapsulation still has data gaps that need to be filled. There are few studies that evaluate and compare the effect of the homogenization process (pressure, cycles) regarding the encapsulation efficiency and lipid oxidation of the fish oil (PUFA) nanoemulsion powders, which are key parameters to produce a stable product during storage. This study examined the impact of sodium caseinate and Tween 80 as emulsifiers and the homogenization parameters on emulsion droplet size in light of the promising properties of fish oil nanoemulsions and the scarcity of studies on its microencapsulation. Additionally, the concentration of various polysaccharides (maltodextrin, sodium alginate, Arabic gum) as wall materials was assessed in relation to the encapsulation efficiency. The physical properties of microcapsules were evaluated through particle size analysis, encapsulation efficiency, color, and Fourier transform infrared spectroscopy (FTIR).

## 2. Results and Discussion

### 2.1. Effect of Homogenization Process Parameters (Pressure, Number of Cycles) on Encapsulation of Fish Oil

#### 2.1.1. Droplet Size and Distribution of Emulsions

The use of high-speed homogenization during the emulsion preparation resulted in emulsions with a mean droplet diameter (MDD) greater than 2.5 μm with a broad distribution (Polydispersity Index, PDI > 0.5), which was insufficient to produce a stable emulsion over time. This initial homogenization step was used to premix the aqueous phase with the continuous oil phase. In order to further reduce droplet size and improve emulsification, additional mechanical energy was required, which was provided through a second step of homogenization, using HPH.

**Figure 1:**
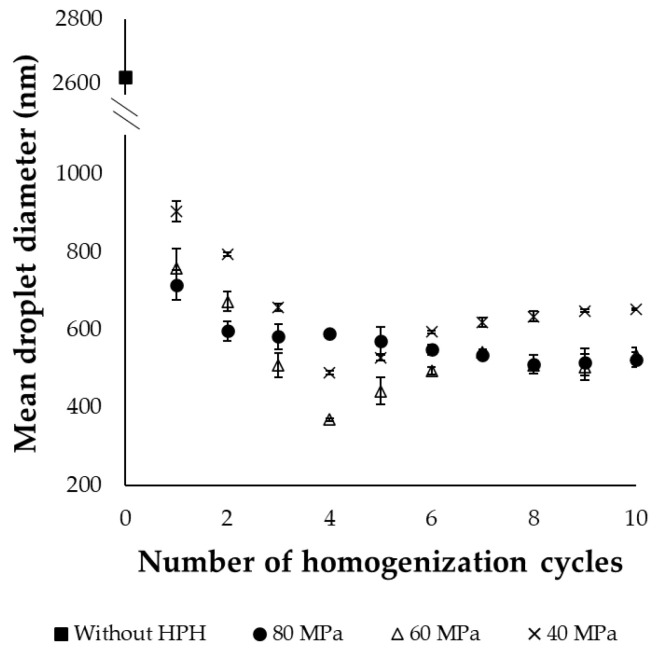
Effect of pressure (40, 60, 80 MPa) and number of passes (from 0 to 10) during high-pressure homogenization on the mean droplet diameter of fish oil-in-water nanoemulsions. Error bars represent the standard deviation of the obtained experimental replicates (n = 3).

The effects of pressure and number of homogenization cycles on MDD are presented in Figure 1. According to the results, both parameters significantly affected the size of the emulsions (*p* < 0.05). Increasing pressure from 40 to 60 MPa led to a significant reduction (*p* < 0.05) in MDD and PDI of the emulsion by up to 25% and 19%, respectively, depending on the number of cycles. Emulsion formation occurs in two stages: first, the droplets of the dispersed phase are disrupted or deformed, increasing the surface area, then emulsifiers stabilize the newly formed droplets by covering the surface area and preventing coalescence of the oil droplets [22]. During the HPH process, the product passes through a tiny gap in the homogenizing valve under high pressure. As a result, the aqueous and oily phases are subjected to intense shear forces and turbulence, leading to the disruption of the dispersed phase (fish oil in this study) into smaller droplets. In general, the higher pressure generates higher turbulence and shear forces, resulting in a more significant reduction in the droplet size [23,24,25]. Regarding PDI, 60 MPa exhibited narrow distribution with uniform droplets, which was expressed through the lower PDI values (up to 19% reduction) compared to 40 MPa. However, when the pressure increased from 60 to 80 MPa, no significant differences in MDD were observed, while PDI was slightly higher. In addition to droplet breakage, the movement of droplets under pressure could lead to coalescence due to frequent collisions between them. Smaller droplets are also thermodynamically less stable and tend to re-coalesce. After each homogenization cycle, either the surfactants will adsorb to the droplet surface, stabilizing them, or the droplets will aggregate, increasing their size. If the emulsifiers adsorption time is longer than the collision time, the interface will not be fully covered, leading to coalescence. In such cases, higher energy input does not necessarily result in the expected reduction in droplet size, and could instead lead to a broader size distribution. In such cases, higher energy input may not result in the expected reduction in droplet size and could instead lead to a broader size distribution [22]. Moreover, the higher pressure may affect the structure of sodium caseinate, the emulsifier used, potentially reducing its emulsifying efficiency [24]. Similar findings have been observed in other studies on the emulsification of different oils using HPH, confirming the existence of a pressure threshold above which no significant differences were observed in MDD [13,23,24,25,26]. In these studies, the threshold was observed between 50 and 100 MPa.

Homogenization cycles were tested up to 10 passes for each pressure level (40, 60, and 80 MPa) and compared to untreated emulsions. During the first four passes, the MDD significantly decreased after each homogenization cycle (*p* < 0.05) at 40 and 60 MPa. Specifically, at 40 MPa, the MDD was reduced to 905.9 ± 25.3, 795.1 ± 4.4, 658.2 ± 9.9, and 490.9 ± 5.2 nm at the end of the 1st, 2nd, 3rd, and 4th cycles, respectively. Similarly, at 60 MPa, the MDD values were 759.2 ± 49.4, 673.3 ± 24.8, 509.6 ± 32.2, and 369.4 ± 3.8 nm, respectively. At 80 MPa, significant differences (*p* < 0.05) were observed between the 1st (716.7 ± 37.4) and the 2nd homogenization cycle (597.3 ± 24.8); however, further increases in the number of passes did not affect this parameter. PDI followed the same trend as MDD, with the lowest PDI achieved at 40 MPa, 60 MPa, and 80 MPa after four, four, and two homogenization cycles, respectively. At the end of the 1st, 2nd, 3rd, and 4th cycle at 40 MPa, the PDI values were 0.668 ± 0.018, 0.528 ± 0.016, 0.357 ± 0.018, and 0.256 ± 0.007, respectively. At 60 MPa the values were 0.652 ± 0.040, 0.322 ± 0.011, 0.233 ± 0.025, and 0.183 ± 0.007, following the 1st, 2nd, 3rd, and 4th cycle, respectively. The reduction in both MDD and PDI could be attributed to the increased energy input during emulsification as the number of cycles increased. However, beyond the fourth cycle, additional homogenization passes did not significantly reduce MDD and PDI; instead, the values remained relatively stable or slightly increased. This behavior has also been reported in other studies [23,25,26,27]. The most likely explanation is that the homogenization process significantly increases the surface area of the fish oil droplet, and beyond a certain point, the available emulsifier molecules may not be sufficient to fully cover the extended oil–water interface formed. Emulsions at 80 MPa exhibited the broadest size distribution, with the values ranging from 0.459 to 0.704, due to the aforementioned coalescence. Keogh et al. [28,29] have also investigated the effects of homogenization pressure (15–50 MPa) and cycles (1–5) on the emulsion size of encapsulated sand eel oil with casein and lactose. Τheir results confirmed that the minimum MDD (0.66 μm) was achieved at the maximum pressure and number of cycles. On the other hand, Drusch [29] concluded that the number of cycles had no significant effect on the emulsion size of encapsulated fish oil, while pressure played a more critical role, with reductions observed when 50 and 10 MPa were applied for the 1st and 2nd homogenization step, respectively.

#### 2.1.2. Encapsulation Efficiency of the Final Dried Product

**Figure 2:**
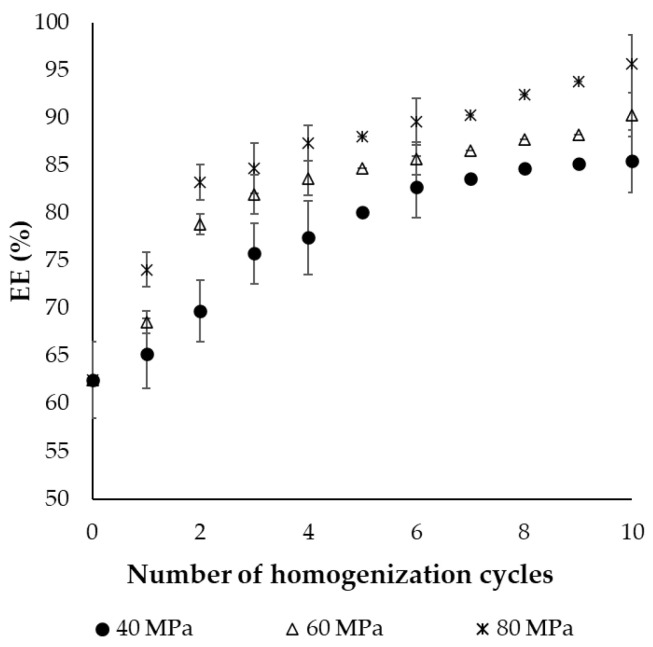
Effect of pressure (40, 60, 80 MPa) and number of passes (from 0 to 10) during high-pressure homogenization on the encapsulation efficiency (EE) of fish oil-in-water nanoemulsions. Error bars represent the standard deviation of the obtained experimental replicates (n = 3).

Encapsulation efficiency indicates the amount of fish oil which is successfully entrapped inside the freeze-dried capsules, which is important for maintaining stability and prolonged storage. The effect of pressure and number of homogenization cycles on the final dried fish oil-filled powders after lyophilization was also assessed, with the encapsulation efficiency (EE) results shown in Figure 2. The lowest pressure (40 MPa) exhibited the lowest EE, reaching a value of 85.5% after 10 cycles of homogenization. The highest EE values achieved at 60 and 80 MPa were equal to 90.3 and 95.6%, respectively. In contrast, EE without the HPH process was only 62.5%, which was insufficient to prevent lipid oxidation and ensure a stable food product. The low encapsulation yield under these conditions is linked to the high amount of fish oil on the surface of the matrix. Surface oil is more susceptible to oxidation, and can also affect the dispersibility and wettability of the final dried product [30]. These findings also highlight the crucial role of HPH in fish oil powders preparation, as evidenced by the significant difference in EE (*p* < 0.05) without and with the process. Increasing the number of passes significantly (*p* < 0.05) improved EE up to the fourth to sixth homogenization cycle, depending on the applied pressure. A further increase in the number of cycles had no significant impact on EE. These results were well corelated to the respective findings for MDD, as smaller oil droplets can be more effectively enclosed by emulsifiers and encapsulating agents, reducing the surface content of non-encapsulated core material and increasing encapsulation yield [11]. Similar results were reported by Keogh et al. [28], who established that the minimum surface fat content (37.5%) was achieved at the highest pressure after five passes. Other studies have optimized HPH conditions for monolayer and multilayer emulsions of cod liver oil. They found that for monolayer emulsions, increasing both pressure and homogenization cycles improved EE. However, for multilayer emulsions, the optimal EE (40.8%) was achieved under intermediate conditions [31].

#### 2.1.3. Lipid Oxidation of Encapsulated Oil

Lipid oxidation in the final dried product was evaluated using the conjugated dienes (K_232_) and p-anisidine value (p-AV) protocols, which assess primary and secondary oxidation products, respectively. K_232_ values in combination with p-AV provide reliable and sufficient information about the extent of oxidation of the encapsulated fish oil and are well-established indicators of primary and secondary lipid oxidation, and require a smaller amount of oil than the typically measured peroxide values for primary oxidation, rendering them suitable for this experimental design and size. Oxidation was measured at selected homogenization cycles (1, 2, 3, 4, 6, 10) based on the results of MDD and encapsulation efficiency results. These were then compared to samples without HPH process. The first four cycles were selected due to significant differences in droplet size and EE, while the ten-cycle conditions represented an extreme homogenization condition, with six passes serving as an optimal intermediate condition, balancing the benefits of enhanced droplet size reduction and satisfactory EE. Although the ten-cycle process may produce fine droplets, additional cycles do not significantly improve the homogenization, and result in increased energy consumption.

**Figure 3:**
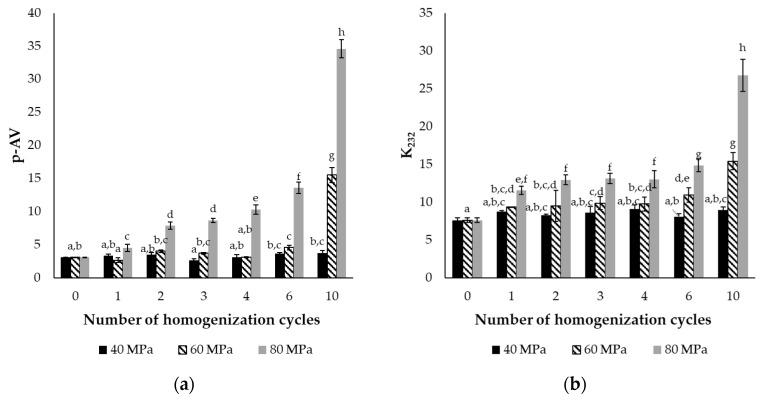
(**a**) p-Anisidine values (p-AV) and (**b**) conjugated dienes of encapsulated fish oil prepared using high-pressure homogenization at different pressures (40, 60, 80 MPa) and cycles of homogenization (0–10). Different superscript letters indicate significant differences (*p* < 0.05) between means of p-AV or K_232_ (±standard deviation of 3 replicates).

According to the results of p-AV and K_232_ (Figure 3), pressure significantly influenced lipid oxidation, with the highest oxidation level being observed at the highest pressure (80 MPa). The initial p-AV and K_232_ values were equal to 3.1 and 7.6, respectively, and after the first cycle at 80 MPa, these values increased by 47.5% and 51.5%, respectively. At 60 MPa, significant differences (*p* < 0.05) were observed after six cycles of homogenization, with maximum p-AV and K_232_ values reaching 15.5 and 15.4, respectively. On the other hand, at 40 MPa, HPH did not affect the oxidation of the encapsulated oil, since the values remained stable even after 10 passes. During homogenization, the applied shear stress caused the temperature of the emulsions to increase, with higher pressure generating greater forces and a more pronounced temperature rise. After the first homogenization cycle at 80 MPa, the temperature exceeded 40 °C, while at 60 MPa, the same temperature was reached after six cycles through the homogenizer. In contrast, the emulsions temperature never exceeded 32 °C at 40 MPa. Temperature is a crucial parameter for lipid oxidation since it accelerates free radical formation and therefore the initiation of oxidation. Unsaturated fatty acids constituted 83% of the total fatty acids in the encapsulated fish oil, and the hydrogen atoms close to the double bonds require low energy for dissociation. Therefore, higher temperatures facilitate hydrogen abstraction, initiating lipid oxidation [32,33]. According to the Codex Alimentarius Commission, the p-AV limit for fish oils intended for human consumption is set at 20 [34]. The p-AV values after homogenization remained below this threshold, except for at 80 MPa with 10 passes. However, the long-term stability of these powders during storage is a main concern. Therefore, 80 MPa is not considered a suitable homogenization condition, since lipid oxidation was already initiated after HPH.

Regarding the effect of pressure on lipid oxidation, Jamshidi et al. also concluded that increasing pressure from 70 to 150 MPa accelerated oxidation, as indicated by TBARs, in double fish oil emulsions W_1_/O/W_2_ [35]. In another study, encapsulated fish oil recovered from cod liver exhibited the lowest TBARs values at moderate pressure conditions (110 MPa) and number of cycles (two cycles) in the case of multilayer capsules. In contrast, monolayer capsules presented significantly higher oxidation levels than the multilayer ones, with the lowest oxidation being observed at the highest pressure (150 MPa) and after either one or three cycles of homogenization [31].

In conclusion, our multi-layer emulsions exhibited enhanced stability, likely due to the lower pressure conditions and the presence of sodium caseinate. These conditions resulted in smaller droplet sizes and higher encapsulation efficiency, which contributed to improved oxidation stability. The reduced droplet size increased the surface area for encapsulation, thereby minimizing the exposure of the fish oil to oxidation.

### 2.2. Effect of Wall Material on Encapsulation of Fish Oil

#### 2.2.1. Encapsulation Efficiency

Along with the emulsification process, selecting the right wall material is essential for creating a stable powder with enhanced encapsulation efficiency that safeguards lipids from oxidation.

According to the results (MDD, EE, lipid oxidation) presented in Section 2.1, 60 MPa and four cycles of homogenization were selected as the optimal conditions for HPH during the preparation of fish oil emulsions. The absence of cryoprotectants in the final dried powder (control samples) resulted in only 40.1% EE. Therefore, the addition of wall material was essential before the freeze-drying process. In this framework, different concentrations of maltodextrin (0–30% *w*/*w* in the feed solution) were tested, in combination with 1% *w*/*w* sodium alginate or 10% *w*/*w* Arabic gum. The use of maltodextrin alone as a wall material proved to be relatively ineffective, as the EE was below 60%. The use of either Arabic gum or sodium alginate without maltodextrin slightly increased the EE (66.2 and 64.8%, respectively), though the values remained low. Maltodextrin alone, without the addition of other biopolymers, cannot be effectively used as wall material due to its insufficient stability during freeze-drying, leading to degradation and loss of encapsulation efficiency. While maltodextrin provides some protection to encapsulated bioactive compounds, it is prone to degradation and loss of structural integrity during the freeze-drying process. Mixtures of polysaccharides and proteins are necessary to prevent ice crystal formation and maintain the structural stability of the encapsulating matrix, thereby enhancing the overall effectiveness of the encapsulation process and preserving the bioactive compounds [36]. These low EE values were in accordance with the low EE of fish oil from sand smelt, sardine, and mackerel by-products, which were encapsulated with maltodextrin (45.6–57.2%) or Arabic gum (47.1–60.6%) [14]. Thus, it was evident that a combination of the cryoprotectants was necessary to form a dense film surrounding fish oil. Table 1 presents the EE of encapsulated fish oil for the different combinations of maltodextrin with the two other polysaccharides. In general, as the concentration of maltodextrin increased, the efficiency of encapsulation was improved. However, no statistically significant differences were observed when the maltodextrin concentration ranged from 20 to 30%. Since the aim of the study was to ensure a high EE of fish oil using the minimum adequate amount of wall material, no further increase in maltodextrin concentration was tested. Higher maltodextrin concentration may not lead to a meaningful enhancement in EE. Moreover, from an economic perspective, increasing the maltodextrin content beyond 30% would lead to higher production costs due to the increased use of wall material without a clear benefit in terms of EE. In addition, excessive maltodextrin addition may alter the functional properties of the final powder, such as solubility, flowability, or sensory characteristics, which could impact its application in food, nutraceutical or other applications. The maximum EE values achieved were 82.9 and 79.1%, when using Arabic gum and sodium alginate, respectively. Moreover, both polysaccharides exhibited similar yields between them at the same maltodextrin concentration. The combination of polysaccharides with sodium caseinate, used as emulsifier, formed a dense and cohesive matrix around the encapsulated fish oil, ensuring the structural stability of the encapsulated material. Proteins and polysaccharides, characterized by high molecular weight and numerous functional groups, can interact with hydrophobic or hydrophilic compounds of the emulsion through electrostatic interactions (Van der Waals forces), hydrogen bonding, and covalent interactions [36,37,38].

In the literature, various components have been evaluated as wall materials and/or emulsifiers for the encapsulation of fish oils. These include fish proteins, other animal-based (e.g., whey protein, sodium caseinate, myofibrillar protein) or plant-based proteins (e.g., zein, soybean, sunflower or rice protein), and polysaccharides (e.g., maltodextrin, inulin, cellulose, chitosan, alginates, Arabic gum, gelatin, carrageenan), or their combinations [39]. Comparable high EE values have also been reported for the encapsulation of cod liver oil using a combination of gelatin and maltodextrin in a ratio of 10:10:30 [40], or using sodium caseinate, maltodextrin, and Arabic gum in a ratio of 1:2:1:1 [41]. Contrary to our findings, Özyurt et al. reported that the combination of sodium caseinate with maltodextrin in different ratios (1:2, 1:1, 2:1) and with a constant fish oil-to-wall material ratio of 1:2 resulted in a significantly higher EE using the spray-drying process (84.5–85.9%) compared to the lower EE observed in this study [42]. Additionally, Charles et al. achieved higher encapsulation yields for tuna oil (83–86%) using maltodextrin and whey protein in 5:1 or 6.5:1 ratios, employing the freeze-drying process [43].

#### 2.2.2. Color of Powders

The visual appearance of encapsulated fish oils, which is strongly related to color measurements, is a critical quality parameter that affects product acceptability by consumers, particularly for food and nutraceutical applications. The L* color parameter represents the lightness of the sample, a* indicates redness or greenness, and b* yellowness or blueness. Additionally, whiteness index was also selected to assess the encapsulated fish oil powders, as it can indicate the presence of surface oil and possible oxidation.

**Figure 4:**
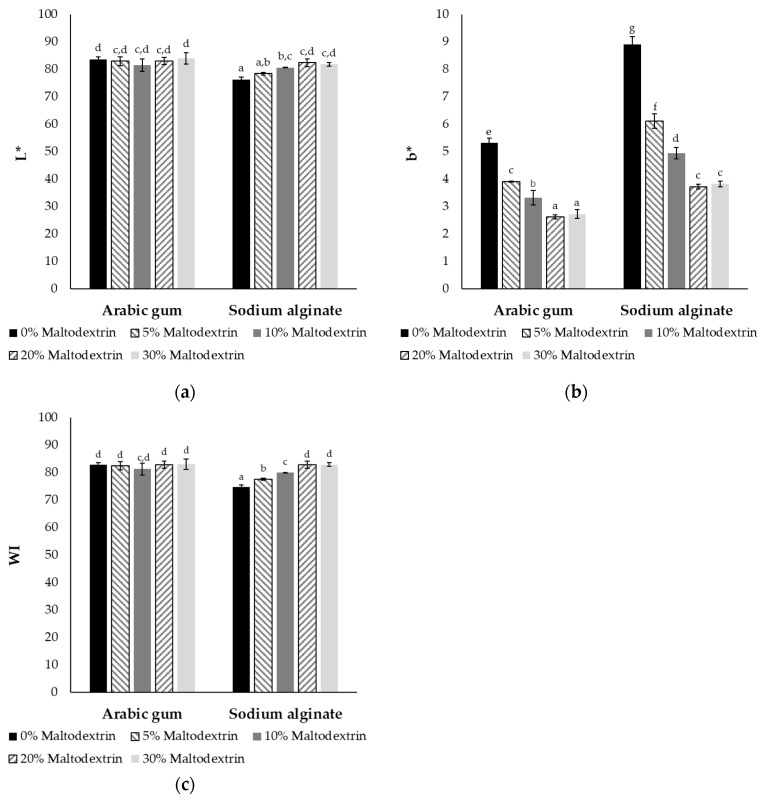
(**a**) L* color parameter, (**b**) b* color parameter, and (**c**) whiteness index of encapsulated fish oil using different concentrations of maltodextrin (0–30% in the feed solution) with the addition of 1% *w*/*w* sodium alginate or 10% *w*/*w* Arabic gum. Different superscript letters indicate significant differences (*p* < 0.05) between means of L*, b* or WI (±standard deviation of 5 replicates).

In Figure 4, the results for the L* and b* parameters, as well as the whiteness index (WI) of the samples with different wall materials are presented. Regarding the a* parameter, values ranged from −0.8 to −1.0, with no statistically significant differences observed in relation to maltodextrin concentration or between sodium alginate and Arabic gum. This is why a* values are not shown in Figure 4. Sodium caseinate, maltodextrin, Arabic gum, and sodium alginate were almost completely white, while fish oil and Tween 80 were characterized by a yellowish hue. Therefore, none of the aforementioned materials could contribute to the greenness or redness of the powders. The lightness of the powders containing Arabic gum ranged from 81.2 to 82.9, with no statistically significant differences observed within the different maltodextrin concentrations. On the other hand, the lightness of powders with sodium alginate increased by up to 8% when maltodextrin concentration increased from 0 to 20%, with no statistically significant differences being noticed between 20 and 30%. Additionally, the lightness of Arabic gum samples was higher compared to sodium alginate at the same concentration of maltodextrin. For both polysaccharides examined, the b* value decreased as maltodextrin concentration increased up to 20%. The WI values exhibited the same trend as the L* and b* parameters, indicating that maltodextrin concentration significantly influenced this parameter. The slightly lower L* values and whiteness along with the higher b* values in sodium alginate could be attributed to its lower concentration (1% *w*/*w* in the feed solution) compared to Arabic gum (10% *w*/*w*). As a result, fish oil made up a higher percentage of the total solids. For instance, in samples without maltodextrin, fish oil represented 52.6 and 35.7% of the total solids in the final dried powders with sodium alginate and Arabic gum, respectively. Moreover, b* values and whiteness showed a strong correlation with the encapsulation efficiency. Higher EE indicates that fewer oil droplets were exposed on the surface, reducing yellowness and leading to more colorless (white) powders, since fish oil naturally has a yellowish hue. The results of this study were in accordance with previous findings on the color of encapsulated fish oils using the same wall materials, in which L* values ranged from 82.5 to 91.0, a* values ranged from −0.03 to −0.6. and b* ranged from 9.6 to 16.5 [41,42,44]. The slightly higher b* values in these studies could be attributed to spray drying, in which the higher processing temperature could cause Maillard reactions between the amino acids and the reducing sugars in the wall materials or emulsifiers. Maillard reaction products result in more yellowish coloration in the final powders [11,44].

#### 2.2.3. FTIR Spectra

**Figure 5:**
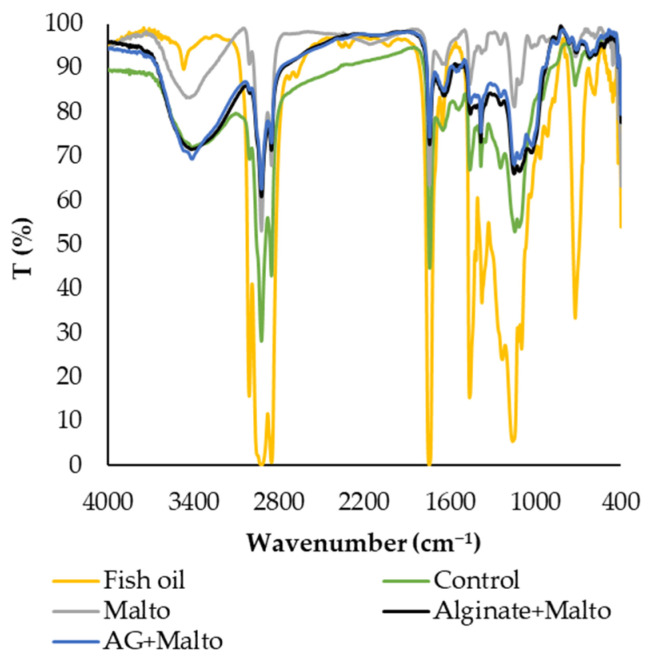
FTIR spectra of non-encapsulated and encapsulated fish oils without (Control) or with different wall materials: maltodextrin (Malto), Arabic gum + maltodextrin (AG + Malto), sodium alginate + maltodextrin (Alginate + Malto).

The FTIR spectra of selected fish oil-filled powders are presented in Figure 5 and compared to the spectral features of non-encapsulated fish oil in order to evaluate the efficiency of encapsulation. The selected samples included a control powder without maltodextrin, Arabic gum, or sodium alginate, as well as powders containing 20% *w*/*w* maltodextrin, either alone (Malto) or combined with Arabic gum (AG + Malto) or sodium alginate (Alginate + Malto) as wall materials. Fish oil mainly consists of monounsaturated and polyunsaturated fatty acids, which are characterized by the presence of double bonds between two carbon (C) atoms. The most intense peaks (corresponding to the lowest transmittance) in the fish oil spectrum were detected at 2920 and 2864 cm^−1^, attributed to the asymmetric and symmetric C–H stretching vibrations of methylene (–CH_2_ groups), which are characteristic of fatty acids. Additionally, a peak at 1746 cm^−1^ was associated with the stretching of C=O bonds in carbonyl groups, while another peak at 1160 cm^−1^ corresponded to the CH_2_ bending in methylene groups. Other characteristic peaks with higher transmittance compared to the aforementioned ones were observed at 3471 cm^−1^ due to O–H stretching, 3011 cm^−1^ due to =C–H stretches of cis alkenes (characteristic of unsaturated fatty acids), 1653 cm^−1^ due to C=C stretching of alkenes or conjugated groups, 1463 cm^−1^ due to C–H bending vibrations (in-plane deformation) of –CH_2_ and –CH_3_ groups, 1376 cm^−1^ due to symmetric bending of –CH_3_ groups, 1232 cm^−1^ due to C–O stretching, 965 and 915 cm^−1^ due to =C–H bending of trans and cis groups, respectively, and at 721 cm^−1^, related to C–H rocking of olefins [45,46].

Most of the aforementioned characteristic bands were less intense and exhibited higher transmittance in the encapsulated fish oils, even in the control samples. The improved encapsulation of fish oil achieved with the use of maltodextrin, sodium alginate, and/or Arabic gum compared to control powders was further confirmed by FTIR spectra. Moreover, extra bands appeared in the FTIR spectra of the encapsulated oils, corresponding to the presence of emulsifiers or wall materials. Peaks observed in the area between 3300 and 3400 nm^−1^ and at 1029 nm^−1^ were attributed to the stretching of O–H bonds and to glycosidic bonds, which are characteristics of Arabic gum, maltodextrin, and sodium alginate molecules. Sodium caseinate, a protein used as emulsifier, exhibited a characteristic band at 1640–1645 nm^−1^ due to C=O stretching vibrations and another peak at 1540 nm^−1^, attributed to C–N stretching and N–H bending [19,47].

## 3. Materials and Methods

### 3.1. Materials

Polyoxyethylene (80) sorbitan monooleate (Tween 80, T80) (HLB = 15), sodium caseinate (CAS), sodium alginate (SA), Arabic gum (AG), petroleum ether, iso-octane, and p-anisidine were purchased from Acros Organics (Fairlawn, NJ, USA). Maltodextrin (MD, Glucidex index 18DE) with a molecular mass of 947 Da was purchased from Astron Chemicals SA (Athens, Greece). Fish oil with 12.8% DHA and 9.1% EPA was recovered as described in [32].

### 3.2. Preparation of Oil-in-Water Nanoemulsion (o/w)

The formulation of the o/w fish oil nanoemulsion was as follows: 8% *w*/*w* of a mixture of casein with Tween 80 (1:1 *w*/*w*) as emulsifier, 10% *w*/*w* fish oil, and 82% *w*/*w* water. Firstly, the aqueous phase was prepared by combining the mixture of CAS:T80 (1:1) with water. The continuous (aqueous phase) and dispersed phase (fish oil) were homogenized (9000 rpm, 10 min) at 40 °C with a high-speed homogenizer (CAT Unidrive 1000, CAT Scientific, Paso Robles, CA, USA). Then, the coarse emulsions were further homogenized in a high-pressure homogenizer (APV 1000; Albertslund, Denmark). High-pressure homogenization (HPH) was carried out at 40, 60, and 80 MPa for 1–10 cycles.

### 3.3. Freeze-Drying Process

The wall material was prepared by mixing maltodextrin at various concentrations (0%, 5%, 10%, 20%, and 30% *w*/*w*) with 10% *w*/*w* Arabic gum, or 1% *w*/*w* sodium alginate. The o/w nanoemulsions were mixed with the wall material at a ratio of 1:1 *w*/*w*, and then they were frozen at −40 °C for 24 h. The samples were freeze-dried in laboratory-scale freeze-drying equipment (Alpha 1-4LD plus, Christ, Osterode, Germany) at −52 °C and *p* = 0.017 mbar for 48 h. The dried dispersion was collected and ground to obtain a fine powder.

### 3.4. Particle Size Distribution

Dynamic light scattering (Zetasizer Nano ZS 2000, Malvern Instruments Ltd., Malvern, UK) was used to measure the particle size and particle distribution o/w nanoemulsions before and after HPH homogenization at each selected pressure (40, 60, and 80 MPa) and for 1–10 cycles.

### 3.5. Encapsulation Efficiency (EE)

The surface oil masses of the fish oil-encapsulated powders were measured by a simple dissolution-evaporation-weighing method. Briefly, 2 g of powder was added to 20 mL of hexane in a 50 mL centrifugal tube, and the mixture was shaken for 2 min. Then, the mixtures were filtered through filter paper (Whatman No. 1). The collected hexane solution was evaporated in a rotary vacuum evaporator (BUCHI 461, Buchi Labortechnik AG, Flawil, Switzerland) at 45 °C and then the sample in the vials was weighed, which corresponded to the surface oil mass of the powder. The total oil (both surface and encapsulated oil) mass of the powders was measured by a simple disruption-dissolution-evaporation-weighing method. Powder (2 g) was added to 80 mL of hexane in a 100 mL cylindrical capped glass container and was magnetically stirred (400 rpm) for 4 h to extract the oil into the hexane phase, and then the supernatant was filtered. The collected hexane solution was evaporated in a rotary vacuum evaporator (BUCHI 461, Buchi Labortechnik AG, Flawil, Switzerland) at 45 °C, and then the samples were weighed, which corresponded to the total oil mass of the powder. The EE was calculated according to Equation (1).

(1) $$\mathrm{EE}\left(\%\right)=\frac{m_{\mathrm{total}\;\mathrm{oil}}-{\;m}_{\mathrm{surface}\;\mathrm{oil}}}{m_{\mathrm{total}\;\mathrm{oil}}}\times 100\%$$

### 3.6. Color Measurement

The color of the encapsulated oil was measured using the X-Rite i1 Pro colorimeter (X-Rite, Grand Rapids, MI, USA), coupled with Eye-One share software v1.4 (X-Rite, Grand Rapids, MI, USA). The measurements were expressed on the CIELab color scale at D50 illumination, and the parameters L*, a*, and b* were determined. L* represents the lightness of the samples, while negative and positive values of a* represent the green and red colors, respectively. In the case of b*, blue and yellow colors correspond to negative and positive b values, respectively. Five replicates were taken for each sample, and then the whiteness index (WI) was calculated using Equation (2):

(2) $$\mathrm{WI}=100-\sqrt{{\left(100-L^{*}\right)}^{2}+{a^{*}}^{2}+{b^{*}}^{2}}$$

where L*, a*, and b* are color parameters.

### 3.7. Oxidation of Fatty Acids

The oxidation level of encapsulated fish oils was determined based on p-anisidine value (p-AV) and conjugated dienes (K_232_). The p-anisidine value was determined by the AOCS official method Cd 18-90 [48]. The K_232_ extinction coefficient was measured after dilution of 0.25 g of extracted oil in 25 mL of 2,2,4-Trimethylpentane (isooctane) and measurement of the absorbance at 232 nm using a spectrophotometer (Unicam Helios, Spectronic Unicam EMEA, Cambridge, UK). The coefficient calculations were based on Equation (3):

(3) $$K_{232}=\frac{A}{C\cdot l}$$

where K_232_ is the extinction coefficient, *A* is the absorbance at 232 nm, *C* is the concentration of oil expressed in g/100 mL isooctane, and *L* is the path length of the cuvette used.

### 3.8. Fourier-Transform Infrared Spectroscopy (FTIR)

The powders were characterized by infrared spectroscopy in the region from 4000 to 400 cm^−1^ using a FT-IR spectrometer (FT-IR/ATR Pro 410-5 Jasco 4200, PerkinElmer Instruments, Norwalk, MS, USA). The received spectra were processed with baseline correction after exclusion of carbon dioxide and moisture transmission.

### 3.9. Statistical Analysis

Data were described as mean value ± standard deviation (n = 3). The main effect analysis of variance (ANOVA) method was used for statistical comparisons at a significance level of 95% (*p*-value < 0.05) based on Duncan test with STATISTICA 12.0 (StatSoft Inc., Tulsa, OK, USA).

## 4. Conclusions

This study underlines the effect of high-pressure homogenization and wall matrix composition on the encapsulation efficiency of fish oil derived from sea bass side streams, offering a wide range of potential applications in food systems. To the best of our knowledge, this is the first study systematically evaluating the impact of pressure and homogenization cycles on fish oil emulsions, while also comparing different types of wall materials. Based on the obtained results, both HPH parameters affected the emulsions and the encapsulation efficiency and oxidation level of freeze-dried fish oil-filled powders. While the highest pressure achieved the highest EE (up to 96%), it also accelerated lipid oxidation even after a single pass. Moderate pressure (60 MPa) emerged as the optimal condition, since it provided balance between efficient encapsulation and oxidative stability, producing emulsions with similar droplet size, slightly lower EE, and reduced oxidation up to four homogenization cycles. These results emphasize the importance of optimizing HPH conditions to avoid excessive oxidative degradation while maintaining high encapsulation efficiency. The effect of different biopolymers (maltodextrin, sodium alginate, Arabic gum) was also evaluated to stabilize the nanoemulsion systems during the freeze-drying process. The combination of maltodextrin with sodium alginate or Arabic gum produced fine powders with enhanced encapsulation yield (up to 83%) and increased whiteness, which is strongly linked to visual appearance and consumer acceptability. Therefore, it is crucial to select the appropriate biopolymers in designing microencapsulated fish oil. The findings of this study provide valuable insights into optimizing fish oil encapsulation strategies, enabling the effective delivery of omega-3 rich fish oils in dehydrated forms. In future studies, it will be valuable to explore release rates, oxidation stability, and how different homogenization conditions or wall materials affect the release of fish oil during simulated (*in vitro*) human digestion to further assess the effectiveness of the encapsulation system.

## Figures and Tables

**Table 1:** Encapsulation efficiency of fish oil powders using different concentrations of maltodextrin (0–30% *w*/*w* in the feed solution) with the addition of 1% *w*/*w* sodium alginate or 10% *w*/*w* Arabic gum.

	Arabic Gum	Sodium Alginate
0% maltodextrin	66.2 ± 2.3	64.8 ± 2.2
5% maltodextrin	72.2 ± 2.1	70.9 ± 2.1
10% maltodextrin	76.5 ± 0.8	75.4 ±0.6
20% maltodextrin	82.4 ± 1.5	77.3 ± 1.7
30% maltodextrin	82.9 ± 1.8	79.1 ± 2.1

Data are expressed as the mean value ± standard deviation of 3 replicates.

## Data Availability

The original contributions presented in the study are included in the article; further inquiries can be directed to the corresponding author.

## Abbreviations

The following abbreviations are used in this manuscript:

EE	Encapsulation efficiency
FTIR	Fourier-transform infrared spectroscopy
CAS	Sodium caseinate
SA	Sodium alginate
AG	Arabic gum
PUFA	Polyunsaturated fatty acids
DHA	Docosahexaenoic acid
EPA	Eicosapentaenoic acid
PDI	Polydispersity Index
WI	Whiteness Index
